# Different expression levels of interleukin-35 in asthma phenotypes

**DOI:** 10.1186/s12931-020-01356-6

**Published:** 2020-04-16

**Authors:** Wei Li, Ruihan Gao, Tong Xin, Peng Gao

**Affiliations:** 1 Department of Respiratory and Critical Care Medicine, The Second Hospital of Jilin University, Changchun, Jilin, 130041 China; 2 Department of Medical Laboratory Technology, Beihua University, Jilin, 132013 Jilin China; 3 Department of Respiratory, Xinqiao Hospital, Army Medical University (Third Military Medical University), Chongqing, 400037 China

**Keywords:** Asthma, IL-35, Induced sputum, Neutrophils, Eosinophils

## Abstract

**Background:**

Interleukin (IL)-35 is a newly discovered inhibitory cytokine which is produced by regulatory B and T lymphocytes and belongs to the IL-12 family. It plays a suppressive role in human inflammatory diseases; however, its role in asthma phenotypes is unclear. Our study focuses on the sputum IL-35 level in patients and investigates different airway inflammation capacities of sputum IL-35 in patients with different asthma phenotypes.

**Objective:**

We aimed to determine the sputum IL-35 levels in asthmatic patients with clinical remission phenotypes and control subjects and to investigate possible correlations among lung function, age, sex, fractional exhaled nitric oxide (FeNO), and smoking history in these phenotypes.

**Methods:**

Sputum samples were collected from patients with clinical asthma remission (*n* = 89, 37 males, age 52.24 ± 13.32 years) and a healthy control group (*n* = 19, 9 males, age 44.58 ± 16.3 years). All subjects underwent sputum induction. Induced sputum was assessed for inflammatory cell count, and sputum levels of IL-35 and other cytokines were measured by ELISA and Cytometric Bead Array, respectively.

**Results:**

Sputum IL-35 (median (q1, q3)) levels showed no significant difference between asthma patients (4.89 ng/mL (2.97, 22.75)) and healthy controls (6.01 ng/mL (4.09, 30.47)). However, the sputum IL-35 level was significantly reduced in patients with eosinophilic asthma (EA) (3.95 ng/mL (2.80, 11.00)) compared to patients with neutrophilic asthma (NA) (40.59 ng/mL (20.59, 65.06), *p* = 0.002), paucigranulocytic asthma (PA) (6.25 ng/mL (3.10, 24.60), *p* = 0.012), and mixed granulocytic asthma (MA) (22.54 ng/mL (2.58, 52.45), *p* = 0.026). IL-35 levels in sputum showed a positive correlation with sputum neutrophil cells and a negative correlation with FeNO, FEV1% predicted, and FVC predicted. Furthermore, sputum IL-35 had a significant positive association with Th1-related factors and a negative correlation with Th2-related factors.

**Conclusions:**

Sputum IL-35 is likely involved in different pathophysiological mechanisms of NA and EA and exerts different effects in asthma phenotypes.

## Background

Over a 25-year period, from 1990 to 2015, the prevalence of asthma increased by 12.6%; and in 2015, a total of 358.2 million people suffered from asthma, including 168 million men and 190.2 million women [1]. Asthma is one of the most common types of bronchial inflammation in respiratory diseases and is clinically characterized by reversible bronchoconstriction and airway hyperresponsiveness (AHR) [2, 3]. Airway inflammation is usually thought to be caused by the Th2 immune response or eosinophil participation, which is a pathogenetic marker of bronchial asthma. However, some patients show neutrophil dominance, have low (or even non-existent) Th2 cytokines, and exhibit a poor response to inhaled corticosteroids [4, 5]. Depending on the type of inflammatory cells present in sputum, asthma can be classified into the following four phenotypes: eosinophilic asthma (EA), neutrophilic asthma (NA), mixed granulocytic asthma (MA), and paucigranulocytic asthma (PA) [6]. In recent years, many studies have shown that each phenotype has a different mechanism of action and a different response to treatment. In EA, biomarkers such as eosinophils [7], periostin, fractional exhaled nitric oxide (FeNO), and IgE [8] can predict corticosteroid reactivity. But for non-eosinophilic asthma, particularly NA, further research on possible biomarkers and treatment strategies is needed [9, 10].

Interleukin (IL)-35 is a newly discovered member of the IL-12 family, which is a heterodimer composed of the p35 and Ebi3 components, and is produced by a variety of cell types and tissues, including T-cells, B-cells, Tregs, monocytes and resting tumor cells [11, 12]. Unlike other IL-12 family members, IL-35 has important inhibitory properties [13, 14]. IL-35 may alter the immune response by inducing proliferation of regulatory T-cells and promoting differentiation of Th17 cells. The cytokines released by Th17 cells induce airway epithelial cells to release chemokines that attract neutrophils. In addition, IL-35 reduces the development of Th2 cells and the production of Th2 cytokines that elevate allergen-specific Th2 responses and allergic airway diseases [15]. In the ovalbumin (OVA)-induced mouse asthma model, treatment with recombinant IL-35 or adenovirus-mediated IL-35 inhibited the extent of AHR and allergic inflammation [15]. Nevertheless, the role of IL-35 in asthma is still controversial. Wong and colleagues found that patients with asthma have higher levels of IL-35 than normal subjects and that higher levels of IL-35 are positively correlated with asthma severity [16]. However, Wang et al. found reduced levels of circulating IL-35 in asthmatic patients and that decreased IL-35 levels increase the number of CD8+ T-cells that produce IL-4 [17].

We hypothesized that the heterogeneity of asthma could lead to different experimental results, as described above. Thus, in this study, we compared the sputum concentration of IL-35 in patients with different asthma phenotypes and investigated the relationship between IL-35 and asthma phenotypes.

## Methods

### Study population

Asthma diagnosis was established according to the guidelines from the American Thoracic Society and was based on current episodic respiratory symptoms (over the past 12 months), clinical diagnosis, and evidence of variable airflow obstruction [18]. Patients were in a stable phase of the disease without taking oral corticosteroids or antibiotics, and there was an absence of worsening of asthma symptoms and treatment changes over the 4 weeks leading up to this assessment.

Matched healthy Chinese volunteers were recruited as normal controls. All control subjects were screened using a questionnaire and a simple physical examination to ensure that they did not show any abnormal signs or history of asthma or other autoimmune diseases.

All study subjects received a written assessment of their clinical evaluation, which included smoking history, respiratory symptoms, and sputum induction. Ethical approval was received from the Ethics Committee of the Second Hospital of Jilin University with the approval number 2014–003.

Exclusion criteria for this study were as follows: pregnant women, severe cardiovascular and cerebrovascular diseases, malignant tumors of various systems and organs, chronic diseases of various systems and organs, active tuberculosis, and interstitial lung diseases.

### Sputum collection

Sputum induction and processing was performed as described in previously published articles [19]. In brief, sputum induction time with hypertonic saline (4.5%) was fixed at 15 min for all participants. For the inflammatory cell count, sputum cells were dispersed using dithiothreitol (DTT), and were resuspended in phosphate buffered saline (PBS, pH 7.4). The suspension (60 μm, Haimen Chunbo Biological Experimental Equipment Factory, China) was then filtered and the total cell count, which included leukocytes and columnar epithelial cells (inflammatory cell count × 0.02/quadrant, TCC), and cell viability (viable cell count/total cell count) were assessed. A cytospin smear was prepared and stained (May-Grunwald Giemsa), and a total of 400 non-squamous cells were counted to classify differential inflammatory cells. The quality of induced sputum samples was assessed; less than 50% of squamous epithelial cells and more than 40% of cell viability were considered significant. The sputum supernatant was stored in a microcentrifuge tube at − 80 °C for subsequent testing.

### Measurement of IL-35 and other cytokines

Quantitative detection of sputum IL-35 was performed using the commercial human interleukin-35 (IL-35) ELISA kit (Catalog Number CSB-E13126h, CUSABIO, China) in accordance with the manufacturer’s protocol. The levels of other cytokines (IL-1β, IL-6, IL-8, IL-10, IL-17A, IL-23, TNF-α, and MCP-1) were measured by Cytometric Bead Array (CBA) under the Multi-Analyte Flow Assay Kit (Biolegend, United States) according to the manufacturer’s instructions. All incubation steps were performed at room temperature and away from the light. Capture beads (75 μL) were added to each titertube and incubated for 2 hours. Then, an antibody detection reagent (25 μL) was added to each titertube, mixed, and incubated for 1 h. The Streptavidin-R-phycoerythrin (SA-PE) detection reagent (25 μL) was then added to each titertube, mixed, and incubated for 30 min. Each titertube was then loaded into a BD LSRFortessa flow cytometer, and a total of 300 events per target were recorded per well. The data were analyzed using the LEGENDplex v8.0 software. IL4 and IL5 were assayed but more than half of the samples were below the lower limit of detection for all sputum inflammation groups (data not shown).

### Asthma phenotype classification

The granulocyte cut-off value of sputum eosinophils was 3%, and the cut-off value of sputum neutrophils was 61% [20]. The patients were divided into EA with sputum eosinophils ≥3%, NA with neutrophils ≥61%, PA with eosinophils < 3% and neutrophils < 61%, and MA with eosinophils ≥3% and neutrophils ≥61%.

### Statistical analysis

All analyses were conducted using Windows Statistical Package for the Social Sciences (SPSS) statistical software, Version 20 (SPSS Inc., IL, USA). Normally distributed data are expressed as the mean ± standard deviation (SD) and were compared using an ANOVA with a least significant difference (LSD) or Student’s t-test. Logarithmic transformation was performed for all levels of inflammatory mediators, and then the normal distribution was examined and analyzed using the variance with an LSD post hoc test. Nonparametric data are reported as the median and interquartile range (IQR) and were analyzed with a Kruskal-Wallis test followed by Bonferroni correction or a Mann-Whitney U test for post hoc analysis. Spearman’s rank correlation coefficient was used to test the correlation and was adjusted for age and BMI. Categorical variables were analyzed using a Chi-squared test. A *p* value of < 0.05 was considered statistically significant.

## Results

### Clinical characteristics of asthma patients and healthy volunteers

There were 89 asthma patients with an average age of 52 years (Table 1). The sputum cell count was available for all asthma patients, and they were all assigned an inflammatory phenotype. Accordingly, 38 (42.7%) asthma patients had EA, 38 (42.7%) had PA, 4 (4.5%) had NA, and 9 (10.1%) had MA.

**Table 1:** Characteristics of patients and healthy controls

Variable	Asthma	Normal	*P* value
Number	89	19	
Age (years)	52.24 ± 13.32	44.58 ± 16.30	0.025
Sex, male (%)	37(41.6)	9 (47.4)	0.617
BMI (kg/m^2^)	23.86 ± 3.49	23.22 ± 3.28	0.589
Ex-smoker, n (%)	23 (25.8)	7 (36.84)	0.667
Smoking index	0 (0,15)	0 (0,5.25)	0.796
Post-FEV1 (L)	2.36 ± 1.00	3.13 ± 0.91	0.009
Post-FVC (L)	3.53 ± 1.20	3.65 ± 0.79	0.677
FeNO (ppd)	42.20 (27.80, 99.65)	22.5 (16.5, 24.00)	0.004
Sputum TCC (10^6^/mL)	3.00 (1.80, 5.40)	2.43 (1.62, 3.24)	0.180
Sputum NEU (10^4^/mL)	10 (1, 91)	0 (0, 6)	< 0.001
Sputum EOS (10^4^/mL)	7 (0, 58)	0 (0, 2)	< 0.001
Sputum MA (10^6^/mL)	1.34 (0.27, 3.52)	2.37 (1.56, 3.12)	0.100
Sputum LY (10^4^/mL)	2.51 (0, 16.46)	5.49 (1.08, 12.96)	0.255

Data are expressed as mean ± SD or median (IQR). Data were analyzed using a Student’s t-test or a Mann-Whitney U test and were adjusted for age. *BMI* Body mass index, *FEV1* Forced expiratory volume in 1 s, *FVC* Forced vital capacity, *FeNO* Fractional exhaled nitric oxide, *TCC* Total cell count, *NEU* Neutrophils, *EOS* Eosinophils, *MA* Macrophages, LY: Lymphocyte

There was no significant difference in gender, body mass index, or smoking index between the asthma group and the healthy control group (*P* > 0.05), but the age of asthma patients was significantly higher than that of healthy controls (*P* = 0.025). The Post-FEV1 in the asthma group was significantly lower than that in the healthy control group (*P* = 0.009). There was a significant increase in FeNO in the asthma group compared with the healthy control group (*P* = 0.004). Compared with the healthy control group, the number of eosinophils and neutrophils was significantly higher in the asthma group (eosinophils: *P* <  0.001, neutrophils: *P* <  0.001) (Table 1). The IL-35 level in the asthma group was lower than that in the healthy control group, but the difference was not statistically significant (*P* = 0.247). IL1β and IL8 were also lower in the asthmatic group compared to the normal control group but not at the level of statistical significance. The levels of IL-6 and MCP-1 in the asthma group were significantly higher than those in the healthy control group (IL-6: *P* = 0.013, MCP-1: *P* = 0.003), while IL-17A showed an opposite trend (*P* < 0.001) (Table 2).

**Table 2:** Sputum chemokine concentrations in asthma patients and healthy controls

Variable	Asthma	Normal	*P* value
Number	89	19	
IL-1β (pg/ml)	37.53 (10.59,151.78)	38.44 (15.7, 72.94)	0.811
IL-6 (pg/ml)	9.43 (3.44, 40.61)	4.36 (2.95, 13.36)	0.013
IL-8 (pg/ml)	3099.66 (278.93, 8805.46)	3428.69 (612.39, 12,145.63)	0.164
IL-10 (pg/ml)	0.78 (0.54, 1.36)	1.35 (0.92, 1.61)	0.052
IL-17A (pg/ml)	6.16 (1.10, 15.09)	53.95 (18.02, 81.59)	< 0.001
IL-23 (pg/ml)	8.10 (3.57, 20.99)	19.33 (8.63, 38.75)	0.034
TNF-α (pg/ml)	0.49 (0.32, 0 .79)	0.46 (0.34, 0.67)	0.404
IL-35 (ng/ml)	4.89 (2.97, 22.75)	6.01 (4.09, 30.47)	0.247
MCP-1 (pg/ml)	118.20 (17.87, 280.02)	25.45 (3.25, 70.32)	0.003

Data are expressed as median (IQR). Data were analyzed using a Mann-Whitney U test and adjusted for age. *IL* Interleukin, *TNF* Tumor necrosis factor, *MCP* monocyte chemoattractant protein

### Clinical features of inflammatory phenotypes in asthma

Gender, BMI, and smoking history were similar among the four asthma subgroups (Table 3). Compared to the other groups, the PA group showed the best lung function, while the EA group was the youngest (Table 3).

**Table 3:** Clinical characteristics and sputum cell numbers in asthma inflammatory phenotypes

Variable	EA	NA	MA	PA	*P* value
Number	38	4	9	38	
Age (years)	47.08 ± 13.54^△ ※^	64.25 ± 6.75	56.22 ± 11.92^§^	55.18 ± 12.18^§^	0.007
Male (%)	15 (39.5)	1 (25.0)	4 (44.4)	17 (44.7)	0.899
BMI (kg/m2)	23.97 ± 3.20	22.44 ± 3.02	25.77 ± 3.74	23.44 ± 3.70	0.271
ACQ† [21]	2.0 (2.0, 3.0)	1.5 (1.0, 2.0)	2.0 (0.3, 2.8)	2.0 (1.0, 2.0)	0.930
ACT† [22]	15 (12, 18)	17.5 (5.5, 19.0)	18 (12, 22.5)	17 (13, 21.75)	0.222
AQLQ† [23]	4 (4, 5)	5 (5, 5)	5 (5, 7)	5 (4, 5)	0.409
Rhinitis (%)†	13 (37.1)	1 (25)	4 (44.4)	17 (44.7)	0.208
Anxiety† [24]	5 (0, 7)	2 (0, 5)	0 (0, 11)	4 (0, 6)	0.473
Depression† [25]	3 (0, 7)	3 (0, 7)	0 (0, 7)	1 (0, 5)	0.771
Ex-smoker (%)†	16 (43.2)	3 (75.0)	6 (66.7)	13 (34.2)	0.154
Smoking index†	0 (0, 6.9)	12.4 (2.4, 33.8)	1.0 (0.0, 32.5)	0.0 (0.0, 19.5)	0.323
Post-bronchodilator FEV1 (L) †	2.33 (2.08, 3.13)	0.67 (0.67, 1.61)	2.27 (1.64, 2.92)	2.42 (1.38, 3.04)	0.538
Post-bronchodilatorFEV1/pred (%)†	87 (70.8, 97.7)	29.3 (27.65, 68.9)	81.4 (57.6, 99.15)	90.8 (54.1, 100.4)	0.634
Post-bronchodilator FVC (L) †	3.74 (3.11, 4.48)	1.9 (1.71, 2.61)	3.44 (3.07, 3.78)	3.67 (2.39, 4.18)	0.892
Post-bronchodilator FVC/pred (%)†	106.67 ± 24.46	73.33 ± 36.35	94.2 ± 24.95	100.59 ± 26.84	0.633
FeNO (ppd) †	42.4 (32.65, 131.5)^△^	23 (18, 76)	72.4 (38, 108.2)	37 (22.7, 47.45) §	0.195
ICS/LABA, n (%)†	37 (97.3)	4 (100)	9 (100)	28 (73.7)	0.239
ICS dose (μg)†	400 (400, 500)	450 (400, 500)	400 (325, 500)	500 (400, 800)	0.319
Sputum Cell Count (median (q1,q3))					
Total cells(10^6^/mL) †	3.6 (1.8, 5.4)	3.0 (0.75, 7.55)	2.9 (1.8, 3.6)	2.9 (1.8, 5.4)	0.812
Neutrophils (10^6^/mL) †	0.07 (0.01, 1.38) ^#※^	2 (0.61, 4.59) ^△§^	1.83 (1.32, 2.65) ^△§^	0.06 (0.01, 0.48) ^#※^	<0.001
Neutrophils, %, †	4.0 (1.2, 10.0) ^#※^	65.25 (61.0, 82.4) ^△§^	70(63.3, 73.4) ^△§^	2.2 (0.6, 24.0) ^#※^	<0.001
Eosinophils (10^6^/mL) †	0.82 (0.21, 1.37) ^#※△^	0.01 (0, 0.13) ^§^	0.12 (0.09, 0.22) ^§^	0 (0, 0.04) ^§^	<0.001
Eosinophils, %, †	17.0 (8.7, 40.1) ^#※△^	1.6 (0.2, 2.8) ^§^	4.9 (3.3, 13.1) ^§^	0.2 (0, 1.0) ^§^	<0.001
Macrophages (10^6^/mL) †	1.55 (0.31, 3.51) ^#※△^	0.56 (0.01, 1.16) ^§※^	0.47 (0.11, 0.77) ^§※^	1.91 (0.56, 4.52) ^§ #※^	<0.001
Macrophages, %, †	58.3 (37.3, 80.5) ^#※△^	15.1 (7.8, 24.8)^§※^	21.3 (11.4, 28.0) ^§※^	85.4 (49.1, 95.6) ^§ #※^	<0.001
Lymphocytes (10^6^/mL) †	0.03 (0, 0.15)	0.21 (0.01, 0.66)	0 (0, 0.01)	0.03 (0, 0.17)	0.406
Lymphocytes, %, †	1.8 (0.5, 6.6)	5.9 (2.9, 8.9)	0 (0, 0.5)	1.4 (0.3, 13.0)	0.562
Columnar epithelial cells (10^6^/mL) †	0.04(0, 1.09)	0.1 (0.02, 1.42)	0 (0, 11.79)	0.05 (0.01, 0.24)	0.072
Columnar epithelial cells, %, †	1.4(0.5, 3.0)^△^	3.8 (2.8, 14.7)	0 (0, 2.6) ^△^	3. 5 (0.8, 11.4) ^§※^	0.017

Note: Data are expressed as mean ± SD or median (IQR). Data were analyzed using an ANOVA or Kruskal-Wallis test. †: adjusted for age. *NA* neutrophil type, *MA* mixed granulocyte type, *PA* granulocyte-deficient type, *EA* eosinophilic type. *p* < 0.05: ^#^ vs.NA, ^※^ vs. MA, ^△^ vs. PA, ^§^ vs. EA. *BMI* Body mass index, *ACT* asthma control test, *ACQ* Asthma control questionnaire, *AQLQ-S* Asthma Quality of Life Questionnaire, *FeNO* Fractional exhaled nitric oxide, *FVC* Forced vital capacity, *FEV1* Forced expiratory volume in 1 s, *ICS* Inhaled corticosteroid, *LABA* long-acting β2-agonists

### IL-35 and other inflammatory mediators in asthma inflammatory phenotypes

**Fig. 1:**
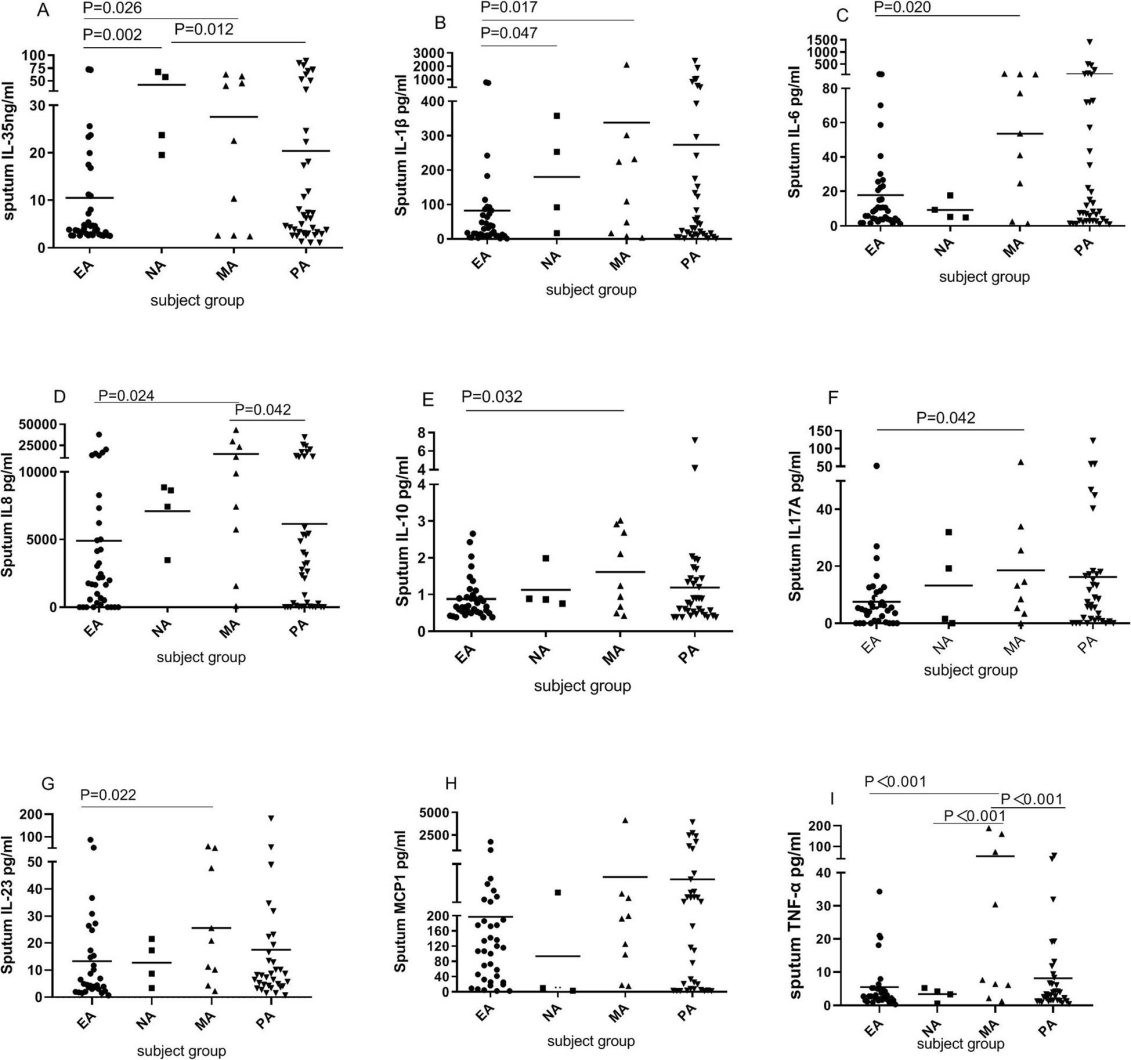
Sputum concentrations of inflammatory mediators in the asthma inflammatory phenotypes

Compared with the PA and MA groups, the sputum IL-35 level was significantly decreased in the EA group and significantly increased in the NA group. Patients with MA had significantly increased concentrations of sputum IL-1β, IL-8, IL-10, IL-17A, IL-23, and TNF-α compared to those in the other groups. Also, the level of IL-6 was significantly higher in the MA group compared with the EA group, but there was no difference among the other asthma inflammatory phenotypes. The sputum MCP-1 level did not differ among the asthma phenotypes, but the level in the MA group was significantly higher than that in the other groups (Fig. 1).

### Association between inflammatory mediators and clinical characteristics

**Fig. 2:**
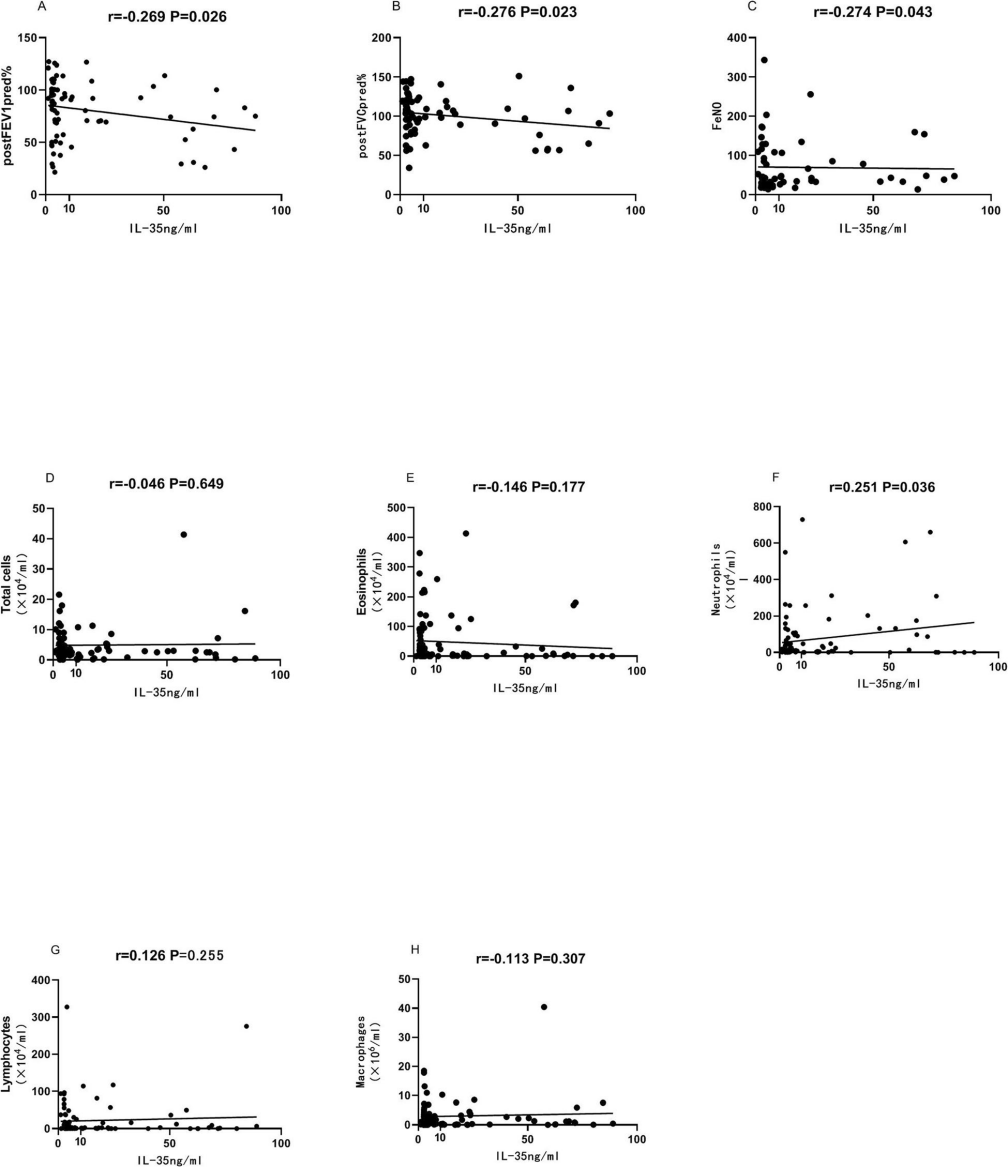
Correlations between sputum IL-35 and clinical characteristics

Sputum IL-35 was negatively correlated with FeNO (ppd), FEV1 (%), and FVC (%) (Fig. 2). Sputum IL-35 was positively correlated with the number of neutrophils (Fig. 2). Sputum IL-35 had a significant positive association with IL-6, IL-8, IL-23, IL-1β and TNF-α (Supplementary Table 1).

### Effect of inhaled corticosteroid (ICS)

We compared the levels of sputum inflammatory mediators in patients taking different doses of ICS. The level of sputum IL-35 was the highest in patients who took a high dose of ICS. However, there was no difference in any of the other mediators between patients who took high-dose ICS and patients who took lower-dose ICS (Table 4).

**Table 4:** Analysis of mediators according to the ICS dose category

Variable (Pg/ml)	Low ICS dose	Middle ICS dose	High ICS dose	*P* value
IL-35 (ng/ml)	3.33 (2.63, 7.40)	6.25 (3.13, 24.16)	6.51 (3.67, 30.63)	NS
IL-1β (pg/ml)	27.28 (6.31, 234.25)	37.53 (12.7, 109.23)	76.57 (7.88, 547.4)	NS
IL-6 (pg/ml)	7.06 (2.93, 62.01)	10.56 (3.99, 30.15)	11.67 (3.67, 104.88)	NS
IL-8 (pg/ml)	2449 (8, 5891)	3248 (558, 11,186)	1861 (54, 9775)	NS
IL-10 (pg/ml)	0.74 (0.51, 1.25)	0.83 (0.52, 1.58)	0.78 (0.53, 1.22)	NS
IL-17A (pg/ml)	5.76 (0.32, 12.24)	6.77 (1.73, 17.12)	5.57 (1.39, 13.7)	NS
IL-23 (pg/ml)	6.43 (3.57, 19.78)	10.11 (3.57, 24.93)	4.89 (2.19, 10.11)	NS
TNF-α (pg/ml)	2.64 (1.34, 6.29)	3.74 (1.7, 7.63)	2.71 (1.87, 16.13)	NS
MCP-1 (pg/ml)	97.8 (5.5, 241.5)	115.8 (16.8, 273.7)	228.3 (98.4, 1062.1)	NS

Data are expressed as median (IQR). Data were analyzed using a Kruskal-Wallis test. *ICS* inhaled corticosteroid, *NS* non-significant, *IL* Interleukin, *TNF* Tumor necrosis factor, *MCP* monocyte chemoattractant protein

## Discussion

Our results show that the sputum concentrations of IL-35 are lower in asthmatics than in healthy controls, which support the results of a previous experiment on IL-35 plasma concentration [26] and a study in children [27]. However, another study evaluated the plasma concentrations of IL-35 by ELISA and found that the IL-35 level in patients with allergic asthma was significantly higher than that in patients with non-allergic asthma [16]. Therefore, we wondered if there was any difference in the sputum IL-35 level in asthma patients or if these dissimilar results can be explained by the heterogeneity of asthma airway inflammation or differences in asthma phenotypes. To address this, we further assessed the different levels of sputum IL-35 in asthma phenotypes. Interestingly, a significantly increased level of IL-35 was found in patients with NA, while a significantly decreased level of IL-35 was noted in patients with EA, compared to patients with other phenotypes. Furthermore, there was a negative correlation between IL-35 and FEV1%, FeNO and a positive correlation between IL-35 and the number of neutrophils and IL-1-related (IL-1β, 6, and 8) inflammatory mediators. These findings suggest that IL-35 may play a key role in the infiltration and activity of eosinophils [28] and neutrophils [29] in asthma and may participate in the airway inflammation mechanism of asthma phenotypes.

The function of IL-35 is complex and the results of different studies may be contradictory. IL-35 has a pro-inflammatory effect in certain diseases, such as rheumatoid arthritis [30, 31] and Lyme arthritis [32], and has an anti-inflammatory effect in other diseases, such as liver inflammation [33], lung inflammation [34], airway inflammation [35], acquired aplastic anemia [36], atherosclerosis [37], arthritis [38], and colitis [39]. IL-35 also exerts different inflammatory effects in different models of the same disease. For instance, the level of IL-35 is significantly increased in collagen-induced arthritis (CIA) animal models showing arthritis symptoms but is reduced in the animal model of arthritis caused by Borrelia burgdorferi [32, 40]. In our research, the level of sputum IL-35 was measured in inflammatory asthma phenotypes and compared with the results of other asthma research. Wong et al. assessed the plasma concentration of IL-35 using ELISA and found that patients with allergic asthma had significantly higher levels of IL-35 than those with non-allergic asthma. Plasma IL-35 concentration displayed a significant positive correlation with the severity of asthma symptoms [16]. However, another study showed the exact opposite result; this work found a decrease in circulating IL-35 levels in asthmatic patients, which increased the number of CD8+ T-cells that produce IL-4 [17]. In an OVA-induced asthma mouse model, adenovirus-mediated IL-35 [41] or recombinant IL-35 [35] was used and inhibited the extent of AHR and allergic inflammation. Mice with glucocorticoid-sensitive eosinophilic airway inflammation induced by a HDM allergen-specific memory/effector Th2 cell line and treated by pVAX-IL-35 DNA via the intranasal route showed reduced allergen-specific airway inflammation compared to controls [15]. In addition, the intramuscular injection of pVAX-IL inhibited circulating allergen specificity and total IgE levels over a longer period of time. These differential findings may have been obtained due to the differences in asthma phenotypes.

Our results suggest that IL-35 may promote neutrophil exudation, which is consistent with previous results. The JM Zou team showed that the neutrophils in tumor tissues of IL-35 mice were significantly higher than those in tumor tissues of naive mice. Consistently, the proportion of neutrophils in blood cells in IL-35 mice was significantly increased compared to that in control mice; thus, indicating that the in vivo expression of IL-35 promotes the mobilization of neutrophils. Based on the expression of various chemokines to neutrophils in the tumor microenvironment, it was suggested that IL-35 could promote the infiltration of neutrophils by increasing the circulating neutrophils [29]. The research by K Kanai et al. showed that airway delivery of IL-35 significantly reduced the number of eosinophils and the production of CCL11 and CCL24 in bronchoalveolar lavage fluid (BALF) from LPS-stimulated EBI3-deficient mice. On the other hand, airway delivery of anti–IL35 significantly increased the number of eosinophils in the BALF from LPS-stimulated WT mice. This study demonstrated that IL-35 inhibits LPS-induced airway eosinophilia, at least in part by reducing the local production of CCL11 and CCL24 [28].

In our study, there was a significant increase in IL-35 level in the sputum of patients with NA. The level of IL-35 in the sputum of asthmatic patients displayed a significant negative correlation with lung function, and a significant positive correlation with IL-1β, 6, and 8, which support the finding of upregulation of sputum IL-35 in patients with more severe NA compared to other asthma types. This observation was further supported by the GS Whitehead and CH Huang study, which found that upregulation of IL-35 was positively correlated with the disease severity score (DSS) [15, 42].

A study found that adenovirus-mediated IL-35 gene delivery can affect allergic airway inflammation and AHR in a mouse asthma model [14, 41, 43]. Based on our results, we suggest that a positive correlation between IL-35 and neutrophils may be associated with severe airway inflammation in asthma. These results may lead to the discovery of different pathophysiological mechanisms of IL-35 in NA and EA.

## Conclusion

Il-35 may participate in the mechanism of the asthma phenotype through various mechanisms. However, more studies are needed to clearly determine the role of IL-35 in the different phenotypes of asthma and to evaluate possible new strategies for therapies based on the IL-35 characterization.

## Supplementary information

**Additional file 1:Supplementary Table 1.** Values of correlation between sputum inflammatory mediators in asthma patients

## Data Availability

All data generated or analyzed during this study are included in this article.

## Abbreviations

IL: Interleukin
TNF: Tumor necrosis factor
ELISA: Enzyme-linked immunosorbant assay
BMI: Body mass index
AHR: Airway hyperresponsiveness
ACQ: Asthma control questionnaire
FeNO: Fractional exhaled nitric oxide
FEV1: Forced expiratory volume in 1 s
FVC: Forced vital capacity
TCC: Total cell count
SD: Standard deviation
ANOVA: Analysis of variance
LSD: Least significant difference
IQR: Interquartile range
OR: Odd ratio
CI: Confidence interval
ICS: Inhaled corticosteroid
COPD: Chronic obstructive pulmonary disease
NA: Neutrophilic asthma
EA: Eosinophilic asthma
PA: Paucigranulocytic asthma
MGA: Mixed granulocytic asthma
