# Supplementary material for: Different expression levels of interleukin-35 in asthma phenotypes
Source: Respir Res. 2020 Apr 16;21:89. doi: 10.1186/s12931-020-01356-6 (PMC7160921; doi:10.1186/s12931-020-01356-6)
Supplement: Supplementary file 1 — Additional file 1:Supplementary Table 1. Values of correlation between sputum inflammatory mediators in asthma patients [file 12931_2020_1356_MOESM1_ESM.doc]

**Supplementary Table 1. Values of c**orrelation between sputum inflammatory mediators in asthma patients

| Variable  (Pg/ml) | IL-35 | IL-1β | IL-6 | IL-8 | IL-10 | IL-17A | IL-23 | TNF-α | MCP-1 |
| --- | --- | --- | --- | --- | --- | --- | --- | --- | --- |
| IL-35 | N/A | 0.378* | 0.392** | 0.534** | NS | NS | 0.288* | 0.320* | NS |
| IL-1β | 0.378* | N/A | 0.664************** | 0.543** | 0.423** | NS | 0.511** | 0.389** | 0.370* |
| IL-6 | 0.392** | 0.664** | N/A | 0.551** | 0.521** | NS | 0.507** | 0.389** | 0.534** |
| IL-8 | 0.534** | 0.543** | 0.551** | N/A | NS | NS | 0.522** | 0.279* | 0.243* |
| IL-10 | NS | 0.423** | 0.521** | NS | N/A | 0.513** | 0.749** | 0.648** | NS |
| IL-17A | NS | NS | NS | NS | 0.513** | N/A | 0.632** | 0.482** | NS |
| IL-23 | 0.288* | 0.511** | 0.507** | 0.522** | 0.749** | 0.632** | N/A | 0.613** | NS |
| TNF-α | 0.320* | 0.389** | 0.389** | 0.279* | 0.648** | 0.482** | 0.613** | N/A | 0.326* |
| MCP-1 | NS | 0.370* | 0.534** | 0.243* | NS | NS | NS | 0.326* | N/A |

The data were analyzed by partial correlation and adjusted for age. **p < 0.001, *p < 0.05, NS: not significant (p > 0.05).
